# Platoon control design for unmanned surface vehicles subject to input delay

**DOI:** 10.1038/s41598-020-80348-4

**Published:** 2021-01-15

**Authors:** Xiaoling Liang, Yuexin Zhang, Guotao Yang

**Affiliations:** 1 Department of Marine Engineering, Dalian Maritime University, Dalian, 116026 China; 2 School of Instrument Science and Engineering, Southeast University, Nanjing, 210096 China

**Keywords:** Mechanical engineering, Information technology

## Abstract

Vessel train formation as a new trend has been raised in cooperative control for multiple vessels. This paper addresses formation control design for a group of unmanned surface vehicles platoon considering input delay. To account for connectivity-preserving and collision-avoiding, Barrier Lyapunov function is incorporated into the constraints design of line-of-sight range and bearing. To alleviate the computational burden, neural dynamic model is employed to simplify the control design and smooth the input signals. Besides, input control arising from time delay due to mechanisms and communication is considered in the marine vessels. Within the framework of the backstepping technique, distributed coordination is accomplished in finite time and the uniformly ultimately boundness of overall system is guaranteed via rigorous stability analysis. Finally, the simulation is performed to verify the effectiveness of the proposed control method.

## Introduction

Future waterborne transportation operates on sea-river, short-sea, and inland waterways by vessel trains. The vessel train consists of a number of vessels including a leading ship and individual ships which can be controlled remotely. The expanding transportation reduces crew, makes optimal use of the existing waterborne transport system and chains up the entire transport into the urban environment. The concept of vessel train formation problem belongs to cooperative control of multiple vessels. Cooperative control strategies for unmanned surface vessels have been an active area in marine engineering. Existing works on cooperative vessels consider the tracking topic, the consensus topic^1,2^, the containment topic^3,4^ and the formation topic. The coordinated tracking has been concerned for a moving leader^5,6^ or leaderless studies^7^ in the practical marine systems. Among the formation control, the leader-follower architecture is an efficient design technique. The leader’s motion guides the other members’ behavior in group along the reference trajectory and the follower in turn serves as the leader in each pair of marine vehicles.

The safety aspects of the vessel train operations should be concerned, otherwise they will have impact on the crew working on board, the other ships operating system even the waterway infrastructure. The command and communication system is critical in navigating and manoeuvring the ships. Connectivity preservation and collision avoidance should be considered, which may destabilize the overall system. Range and bearing constraints are considered for a group of underactuated surface vessels (USVs)^8^. Barrier Lyapunov function (BLF) is proposed to cope with formation tracking for USVs subject to maximum communication and minimum avoidance range^9^. Distributed consensus is dealt with a nonlinear transformation function to achieve connectivity-preserving^10^. However, these methods mentioned above are all developed for USVs. Using prescribed performance control methodology to maintain a desired line-of-sight (LOS) range, the vehicular platoon proceeds along a given trajectory and the internal stability of closed-loop systems can be guaranteed by adaptive formation control of a string of fully actuated surface vessels^11^. However, prescribed performance control easily leads to actuator saturation. BLF can be extended to handle each marine vessel’s relative distance in the platoon.

BLF is developed with the frame of backstepping design. Backstepping scheme suffers from the repeated differentiation of virtual control inputs and further leads to a so-called explosion of complexity. Dynamic surface control has been proposed by introducing the first-order filter to avoid the computational complexity of mathematical operations^12,13^. Command filter is constructed to approximate the derivatives of the command inputs and relieve the computational burden^14–16^. Bioinspired neurodynamics is another alternative approach to deal with the differential explosion problem, which can not only avoid the differential operations of the virtual control inputs but also limit the outputs within a certain range. The input signals pass through the neural dynamic model can trend to a certain range and the attenuation rate can also be adjusted by choosing proper parameters^17^.

On the other hand, the presence of input time delay is a common problem in practical formation systems, which may cause poor performance and instability of the control system. Time delay encountered in input control may arise from the activation of the mechanisms and the communication. Multiple marine vessel systems with time delay are of both theoretical and practical importance. Extensive research has been conducted with controller design of multiagnet systems in the past years. For example, an adaptive finite-time containment control was proposed for nonlinear multiagent systems with input delay^18^. A distributed controller was developed for each agent to track the target in the presence of input delay^19^. Sufficient conditions for mean square consensus were addressed for the cases with input delay in the leader-follower stochastic multi-agent systems^20^. The path-following coordination was dealt with time delay as determined by the communication topology^21^. Robust synchronization of multiple marine vessels was designed by introducing a constant time delay to the communication process^22^. Existing work on marine vessels with input delay is seldom studied, especially for multiple marine vessels.

In the light of these challenges, leader-follower trailing is constructed in resolving the three aforementioned aspects related to waterborne platoon. The idea of this work is to design a control method for a group of vehicles based on local information exchange to achieve a coordinated manner. The highlights of the proposed control are summarized as follows,

- The LOS range and bearing angle of decentralized leader-follower formation control have been restricted by BLF to meet the safety specification and operation performance.
- For the sake of computational simplicity, bioinspired neurodynamics is employed to avoid the derivatives of virtual control inputs. The output signals of the bioinspired model are bounded in a finite interval and smooth without any sharp jumps even actual inputs have sudden changes.
- The marine vehicles in the platoon are subjected to input delay. Artstein model is used as a predictor-like controller to deal with input delay in linear system. However, marine vessel models consist of nonlinear dynamics and uncertain nonlinear functions. Combined with tracking errors limitations, the nonlinear system with input time delay is converted into a delay-free system based on Artstein model.

The rest of the paper is organized as follows. In “Problem description and preliminaries” section, the problem is formulated and the preliminaries are introduced. “Platoon formation control design” section presents the controller design for connectivity preservation and collision avoidance, and the stability of the closed-loop system in the presence of input delay is rigorously analyzed. “Simulation results” section are shown. Lastly, the paper is concluded.

## Problem description and preliminaries

### Problem description

**Figure 1:**
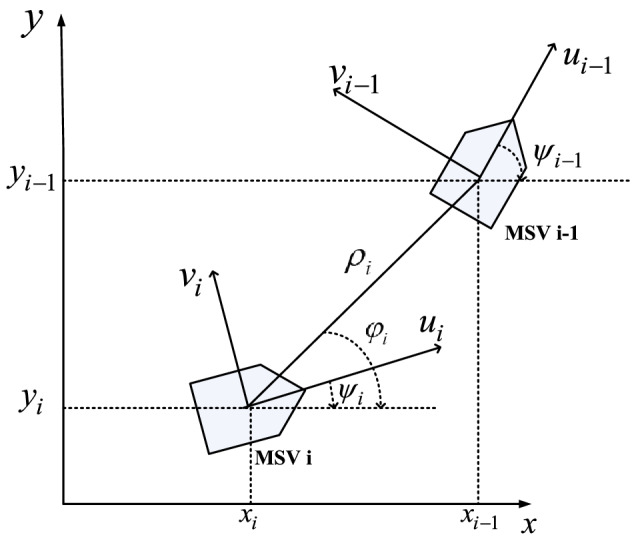
MSVs formation configuration.

Consider a group of marine surface vehicles consisting of a leader and *N* followers. The formation architecture in a pair of leader-follower is shown in Fig. 1. The coordinate frames $\{I\}$ and $\{B\}$ represent the inertial frame and body-fixed frame. The kinematics and dynamics of the *i*-th marine surface vessel (MSV) can be modeled as follows^23^

1 $$\begin{aligned}&{\dot{\eta _i}} = {J_i}( {{\psi _i}} ){\upsilon _i} \end{aligned}$$

2 $$\begin{aligned}&{M_i}{\dot{\upsilon _i}} +{ {C_i}( {{\upsilon _i}} ){\upsilon _i} +{D_i}( {{\upsilon _i}} ){\upsilon _i} +g_i= {{d}_i} + {\tau _i(t-t_d)}} \end{aligned}$$

where ${\eta _i} = {\left[ {{x_i},{y_{i,}}{\psi _i}} \right] ^{\mathrm {T}}}$, ${x_i}$ and ${y_{i}}$ denote the positions of the MSV in $\{I\}$, $\psi _i$ is the yaw angle of the MSV in $\{I\}$, ${\upsilon _i} = {\left[ {{u_i},{\nu _i},{r_i}} \right] ^{\mathrm {T}}}$, ${u_i}$, ${\nu _i}$ and ${r_i}$ represent surge, sway and yaw velocities in $\{B\}$, $M_i$ is the inertia matrix, $C_i$ denotes the matrix of Coriolis and centripetal, $D_i$ is the damping matrix, $g_i$ represents the restoring force vector, ${{d}_i}$ is the vector of external disturbances induced by wind, wave, and ocean currents, etc, ${\tau _i(t-t_d)}$ denotes the control vector of the MSV with time delay, $t_d$ is the delayed time, ${J_i}\left( {\psi _i}\right)$ is the Jacobian transformation matrix,

3 $$\begin{aligned} {J_i}\left( {{\psi _i}} \right) = \left[ {\begin{array}{*{20}{c}} {\cos \left( {{\psi _i}} \right) }&{}\quad { - \sin \left( {{\psi _i}} \right) }&{}\quad 0\\ {\sin \left( {{\psi _i}} \right) }&{}\quad {\cos \left( {{\psi _i}} \right) }&{}\quad 0\\ 0&{}\quad 0&{}\quad 1 \end{array}} \right] \end{aligned}$$

The inertia matrix ${M_i}$ is symmetric positive definite,

4 $$\begin{aligned} {M_i} = \left[ {\begin{array}{*{20}{c}} {{m_{11i}}}&{}\quad 0&{}\quad 0\\ 0&{}\quad {{m_{22i}}}&{}\quad {{m_{23i}}}\\ 0&{}\quad {{m_{32i}}}&{}\quad {{m_{33i}}} \end{array}} \right] \end{aligned}$$

where ${m_{11i}} = {m_i} - X_{\dot{u}i}$, $m_{22i} = {m_i} - {Y_{\dot{v}i}}$, ${m_{23i}} = {m_{32i}} = {m_i}{x_{gi}}- {Y_{\dot{r}i}}$, ${m_{33i}} = {I_{zi}} - {N_{\dot{r}i}}$, $m_{i}$ is the mass of the MSV, ${I_{zi}}$ represents the inertia moment in $\{B\}$, $x_{gi}$ denotes the vessel center of gravity in $\{B\}$, $X_{\dot{u}i}$ is the added mass in surge, $Y_{\dot{v}i}$ and $Y_{\dot{r}i}$ are the added mass in sway, and $N_{\dot{r}i}$ is the added mass in yaw.

The Coriolis and centripetal matrix satisfies ${C_i}=-{C^T_i}$, which is described as

5 $$\begin{aligned} {C_i}( {{\upsilon _i}} ) = \left[ {\begin{array}{*{20}{c}} 0&{}\quad 0&{}\quad { - {m_{22i}}{v _i} - {m_{23i}}{r_i}}\\ 0&{}\quad 0&{}\quad {{m_{11i}}{u_i}}\\ {{m_{22i}}v_i + m_{23i}{r_i}}&{}\quad { - m_{11i}u_i}&{}\quad 0 \end{array}} \right] \end{aligned}$$

The damping matrix $D_i$ is given by

6 $$\begin{aligned} {D_i}({\upsilon _i}) = \left[ {\begin{array}{*{20}{c}} {{d_{11i}}}&{}\quad 0&{}\quad {0}\\ 0&{}\quad {{d_{22i}}}&{}\quad {{d_{23i}}}\\ 0&{}\quad {{d_{32i}}}&{}\quad {{d_{33i}}} \end{array}} \right] \end{aligned}$$

where

7 $$\begin{aligned}&{d_{11i}}\left( {{u_i}} \right) = - ( X_{u_i} + X_{\left| {u_i} \right| u_i}\left| {u_i} \right| +X_{u_iu_iu_i}u_i^2) \end{aligned}$$

8 $$\begin{aligned}&{d_{22i}}({v_i},{r_i}) = - ( Y_{v_i} + Y_{\left| v_i \right| v_i}\left| v_i \right| + Y_{\left| r_i \right| v_i}\left| r_i \right| ) \end{aligned}$$

9 $$\begin{aligned}&{d_{23i}}({v_i},{r_i}) = - ( Y_{r_i} + Y_{\left| v_i \right| r_i}\left| v_i \right| + Y_{\left| r_i \right| r_i}\left| r_i \right| ) \end{aligned}$$

10 $$\begin{aligned}&{d_{32i}}({v_i},{r_i})= - ( N_{v_i} + N_{\left| v_i \right| v_i}\left| v_i \right| + N_{\left| r_i \right| v_i}\left| r_i \right| ) \end{aligned}$$

11 $$\begin{aligned}&{d_{33i}}({v_i},{r_i})= - ( N_{r_i} + N_{\left| v_i \right| r_i}\left| v_i \right| + N_{\left| r_i \right| r_i}\left| r_i \right| ) \end{aligned}$$

with the hydrodynamic damping coefficients $$X_{u_i}$$, $$X_{\left| {u_i} \right| u_i}$$, $$X_{u_iu_iu_i}$$, $$Y_{v_i}$$, $$Y_{\left| v_i \right| v_i}$$, $$ Y_{\left| r_i \right| v_i}$$, $$Y_{r_i} $$, $$Y_{\left| v_i \right| r_i}$$, $$ Y_{\left| r_i \right| r_i}$$, $$N_{v_i}$$, $$N_{\left| v_i \right| v_i}$$, $$N_{\left| r_i \right| v_i}$$, $$ N_{r_i}$$, $$N_{\left| v_i \right| r_i}$$, $$ N_{\left| r_i \right| r_i}$$.

#### **Assumption 1**

The desired reference trajectory is $\eta _{0}=[x_{0},y_{0},\psi _{0}]^{\mathrm {T}}$, whose first time derivative ${\dot{\eta }}_{0}$ is bounded .

#### **Assumption 2**

There exists bounded constants $\overline{d_i}$ for the disturbance term $d_i, i=0,1,\ldots ,N$ of each vessel.

#### **Assumption 3**

The inertia matrix $M_i$ is inverse and we assume $||M^{-1}_i||\le \overline{M_i^{-1}}$ with constant bound $\overline{M_i^{-1}}$.

In this section, the formation objective of this paper is to design control laws such that each marine vessel modeled by (1) and (2) can follow its leader and do not violate the collision and connectivity in the platoon configuration when subject to input time delay. All signals in the closed-loop system can be guaranteed to be bounded during the whole operation.

The platoon formation objectives in this paper are to ensure that

- The connectivity preservation and collision prevention are satisfied on the LOS range and bearing angle schemes between two consecutive MSVs.
- The effect of input delay is considered and the stability is analyzed in constrained platoon control.
- A string of MSVs can achieve the formation tracking.

### Preliminaries

#### **Lemma 1**

^24^ *For any constant* $x \in {{\mathbb {R}}}^n$, *there exists a constant* *k* *satisfying* $\left| x \right| < k$ *such that*

12 $$\begin{aligned} \ln \frac{{{k^2}}}{{{k^2} - {x^2}}} \le \frac{{{x^2}}}{{{k^2} - {x^2}}} \end{aligned}$$

#### **Lemma 2**

^25^ *For bounded initial conditions, if there exists a continuous and positive definite Lyapunov function* *V*(*x*) *satisfying* $v_1(||x||)\le V(x) \le v_2(||x||)$, *such that* ${\dot{V}}(x) \le -\alpha V(x)+\beta$, *where* $v_1$, $v_2$: ${\text {R}}^n \rightarrow {\text {R}}$ *are class K functions and* $\alpha , \beta >0$, *then the solution* *x*(*t*) *is uniformly bounded.*

### Bioinspired model

To describe the behavior of individual neuron, Hodgkin and Huxley firstly proposed a membrane model based on extensive experiments^26^. The model on the voltage characteristic of a cell membrane is constructed as

13 $$\begin{aligned} C_m\frac{dV_m}{dt}=-(E_p+V_m)g_p+(E_{Na}-V_m)g_{Na}-(E_k+V_m)g_k \end{aligned}$$

where $$C_m$$ represents the membrane capacitance, $$V_m$$ is the voltage of the neuron. The parameters $$E_k$$, $$ E_{Na}$$, and $$ E_p$$ are the Nernst potentials for potassium ions, sodium ions, and passive leak current in the membrane, respectively. The functions $$g_k$$, $$g_{Na}$$, and $$g_{p}$$ denote the potassium conductance, the sodium conductance, and the passive channel, respectively.

Grossberg derived the biologically inspired neurodynamic model to describe an online adaptive behavior of individuals^27^. The simplified shunting equation is obtained as

14 $$\begin{aligned} \dot{V}=-AV+(B-V)S(t)^{+}-(D+V)S(t)^{-} \end{aligned}$$

where *V* is the neural activity (membrane potential) of the neuron. Parameters *A*, *B*, and *D* are nonnegative constants, namely, the passive decay rate, the upper and the lower bounds of the neural activity, respectively. The variables $S(t)^{+}$ and $S(t)^{-}$ represent the excitatory and inhibitory inputs, respectively^28–30^. The bioinspired model can be regarded as a low-pass filter. This method can achieve satisfactory tracking performance due to shunting characteristics. The outputs are restricted to a bounded interval and the signals obtained are smooth and continuous.

## Platoon formation control design

As shown in Fig. 1, the relative distance, ${\rho _i}$, between each pair of MSVs and LOS range, ${\varphi _i}$, are defined as

15 $$\begin{aligned} \ {\rho _i}= & {} \sqrt{{{( {{x_{i - 1}} - {x_i}} )}^2} + {{( {{y_{i - 1}} - {y_i}} )}^2}} \end{aligned}$$

16 $$\begin{aligned} \ {\varphi _i}= & {} \arctan 2 ( y_{i - 1} - {y_i},x_{i - 1} - {x_i} ) \end{aligned}$$

The tracking errors of the MSVs are defined as

17 $$\begin{aligned} \ \begin{array}{l} {e_{\rho i}}= {\rho _i} - {\rho _{i,des}}\\ {e_{\psi i}} = {\psi _{i - 1}} - {\psi _i} \end{array}\ \end{aligned}$$

where ${\rho _{i,des}}$ is the desired LOS range.

To avoid collision and connectivity maintenance among vehicles, the desired distance during the whole moving process must satisfy the following equation

18 $$\begin{aligned} 0<\ {\rho _{i,\min col}} < {\rho _i} \le {\rho _{i,\max com}} \end{aligned}$$

where ${\rho _{i,\min col}}$ and ${\rho _{i,\max com}}$ represent the minimum safety distance and maximum effective communication distance respectively. For convenience, we define the minimum and maximum distance errors as

19 $$\begin{aligned} {{\underline{e}}_{\rho i}}=&{\rho _{i,\min col}}-\rho _{i,des}\nonumber \\ {{\bar{e}}_{\rho i}} =&{\rho _{i,\max com}} - { \rho _{i,des}} \end{aligned}$$

Then the constraints of the LOS range errors become

20 $$\begin{aligned} {{{\underline{e}}_{\rho i}}}< {e_{\rho i}} < {{{\bar{e}}}_{\rho i}} \end{aligned}$$

The constraints of the yaw angle errors have similar property as

21 $$\begin{aligned} {{\underline{e}}_{\psi i}}< {e_{\psi i}} < {{{\bar{e}}}_{\psi i}} \end{aligned}$$

where ${{{\bar{e}}}_{\psi i}}$ and ${{{\underline{e}}}_{\psi i}}$ are denoted as the maximum and minimum bounds of yaw angle errors.

*Step 1*: Define the tracking error as

22 $$\begin{aligned} z_{1i} = [ z_{11i},z_{12i} ]^{\mathrm {T}} = {[ {{e_{\rho i}},{e_{\psi i}}} ]^{\mathrm {T}}} \end{aligned}$$

Consider the symmetric barrier Lyapunov function candidate as

23 $$\begin{aligned} \ {V_{1i}} = \frac{1}{2}\ln \frac{{k_{ai}^2}}{{k_{ai}^2 - e_{\rho i}^2}} + \frac{1}{2}\ln \frac{{k_{bi}^2}}{{k_{bi}^2 - e_{\psi i}^2}} \end{aligned}$$

where ${k_{ai}}$ and ${k_{bi}}$ are positive constants satisfying the inequalities $| e_{\rho i} |< k_{ai},| e_{\psi i}| < k_{bi}$, respectively. The time derivative of ${V_{1i}}$ yields

24 $$\begin{aligned} {\dot{V}_{1i}} = \frac{{{e_{\rho i}}{{\dot{e}}_{\rho i}}}}{{k_{ai}^2 - e_{\rho i}^2}} + \frac{{{e_{\psi i}}{{\dot{e}}_{\psi i}}}}{{k_{bi}^2 - e_{\psi i}^2}} \end{aligned}$$

According to Eq. (17), the time derivatives of ${e_{\rho i}}$ and ${e_{\psi i}}$ are given by

25 $$\begin{aligned} {{\dot{e}}_{\rho i}}= & {} - u_i\cos \left( {{\psi _i} - {\varphi _i}} \right) +{{\dot{y}}_{i - 1}}\sin {\varphi _i} + v_i\sin \left( {{\psi _i} - {\varphi _i}} \right) +{{\dot{x}}_{i - 1}}\cos {\varphi _i} \end{aligned}$$

26 $$\begin{aligned} {{\dot{e}}_{\psi i}}= & {} {{{\dot{\psi }} }_{i - 1}} - {{{\dot{\psi }} }_{i }} \end{aligned}$$

*Step 2*: The stabilizing function ${\alpha _i} = [\alpha _{1i},\alpha _{2i},\alpha _{3i}]^{\mathrm {T}}$ is designed as follows

27 $$\begin{aligned} \alpha _{1i}=&\cos (\psi _i-\varphi _i)[k_{di}e_{\rho i}(k_{ai}^2-e_{\rho i}^2) + {{\dot{x}}_{i - 1}}\cos {\varphi _i} +{{\dot{y}}_{i - 1}}\sin {\varphi _i}] \end{aligned}$$

28 $$\begin{aligned} \alpha _{2i}=&-\sin (\psi _i-\varphi _i)[k_{di}e_{\rho i}(k_{ai}^2-e_{\rho i}^2)+ {{\dot{x}}_{i - 1}}\cos {\varphi _i}+{{\dot{y}}_{i - 1}}\sin {\varphi _i}] \end{aligned}$$

29 $$\begin{aligned} \alpha _{3i}=&k_{\psi i}e_{\psi i}({k_{bi}^2 - e_{\psi i}^2})+{\dot{\psi }}_{i-1} \end{aligned}$$

where $k_{di}$ and $k_{\psi i}$ are positive constants.

To avoid the complicated math operations on the derivative of ${\alpha }_{i}$, let ${\alpha }_{i}$ pass through a neural dynamic model and substitute ${\alpha }_{ci}=[\alpha _{c1i},\alpha _{c2i},\alpha _{c3i}]^{\mathrm {T}}$ with ${\alpha }_{i}$ in the following backstepping design. The bioinspired neurodynamics is adopted to smooth the virtual velocity control variables and obtain their derivatives. The neural dynamic model is constructed as^28–30^

30 $$\begin{aligned} \dot{{\alpha }}_{ci}=-A_i{\alpha }_{ci}+(B_i-{\alpha }_{ci})f({\alpha }_{i}) -(U_i+{\alpha }_{ci})g({\alpha }_{i}) \end{aligned}$$

with

31 $$\begin{aligned} f({\alpha }_{i}) = \left\{ {\begin{array}{*{20}{c}} {{\alpha }_{i},}&{}\quad {{\alpha }_{i} \ge 0}\\ {0,}&{}\quad {{\alpha }_{i} < 0} \end{array}} \right. , g({\alpha }_{i}) = \left\{ {\begin{array}{*{20}{c}} { - {\alpha }_{i},}&{}\quad {{\alpha }_{i} \le 0}\\ {0,}&{}\quad {{\alpha }_{i} > 0} \end{array}} \right. \end{aligned}$$

where ${\alpha }_{ci}$ is the output of the neural dynamic model, $A_i$, $B_i$ and $U_i$ are positive parameters, which can be chosen to adjust the attenuation rate. The output can be limited within the region $[-U_i, B_i]$. Then define the error $z_{\alpha i}$ as

32 $$\begin{aligned} z_{\alpha i}={\alpha }_{ci}-{\alpha }_{i} \end{aligned}$$

Based on Eq. (30) with $B_i=U_i$, it results in

33 $$\begin{aligned} z_{\alpha i}=-A_{fi}{\alpha }_{ci}+B_i{\alpha }_{i}-{\dot{\alpha }}_{i} \end{aligned}$$

where $$A_{fi}=A_{i}+f({\alpha }_{i})+g({\alpha }_{i})>0$$.

Let $z_{2i}=[z_{21i},z_{22i},z_{23i}]^{\mathrm {T}}$, then the following error is defined as

34 $$\begin{aligned} {z_{2i}} =[z_{21i}, z_{22i},z_{23i}]^{\mathrm {T}}={\alpha _{ci}}-{\upsilon _i} \end{aligned}$$

Then substituting (25)–(34) into (24), ${\dot{V}}_{1i}$ yields

35 $$\begin{aligned} {{\dot{V}}_{1i}} = - {k_{di}}e_{\rho i}^2 - {k_{\psi i}}e_{\psi i}^2 + z_{\alpha i}^{\mathrm {T}}\Theta _{1i}-z_{2 i}^{\mathrm {T}}\Theta _{1i} \end{aligned}$$

where

$$\begin{aligned} \Theta _{1i}=&\frac{{{e_{\rho i}}\left( { - \cos \left( {{\psi _i} - {\varphi _i}} \right) +\sin \left( {{\psi _i} - {\varphi _i}} \right) } \right) }}{{k_{ai}^2 - e_{\rho i}^2}}+\frac{{{e_{\psi i}}}}{{k_{bi}^2 - e_{\psi i}^2}}. \end{aligned}$$

The dynamic of $z_{2i}$ yields

36 $$\begin{aligned} {M_i}{{\dot{z}}_{2i}} =&{C_i}( {{\upsilon _i}} ){\upsilon _i} + {D_i}( {{\upsilon _i}} ){\upsilon _i} +g_i- {{d}_i}+{M_i}{{\dot{\alpha }}_{ci}}+{M_i}{{\dot{z}}_{\alpha i}} \end{aligned}$$

To compensate the input delay, an auxiliary state $S_i \in {{\mathbb {R}}}^{3 \times 1}$ is defined as following

37 $$\begin{aligned} S_i=z_{2i}-M^{-1}_i\int ^{t}_{t-t_d}{\tau _i}(\theta ){\text {d}}\theta -M^{-1}_iz_{fi} \end{aligned}$$

In (37), $z_{fi} \in {{\mathbb {R}}}^{3 \times 1}$ satisfies the following adaptive law

38 $$\begin{aligned} {\dot{z}}_{fi}=K_{2i}S_i-\Gamma _{1i}z_{2i}-\Theta _i z_{fi} \end{aligned}$$

where $K_{2i}, \Gamma _{1i}, \Theta _i \in {{\mathbb {R}}}^{3 \times 3}$ are constant matrices. Multiply both sides of Eq. (37) by $M_i$, the term of $M_i{\dot{S}}_i$ gives

39 $$\begin{aligned} M_i\dot{S_i}=&M_i{\dot{z}}_{2i}-\tau _i(t)+\tau _i(t-t_d)-{\dot{z}}_{fi}\nonumber \\ =&M_i{\dot{\alpha }}_{ci}+C_i(\upsilon _i)\upsilon _i+D_i(\upsilon _i)\upsilon _i+g_i-d_i- K_{2i}S_i+\Theta _i z_{fi}+\Gamma _{1i}z_{2i}-\tau _i(t)+{M_i}{{\dot{z}}_{\alpha i}}\nonumber \\ =&M_i{\dot{\alpha }}_{ci}+M_{si}-d_i+N_{ci}-\tau _i(t)-K_{2i}S_i -(S^{\mathrm {T}})^{+}{\dot{S}}^{\mathrm {T}}_iz_{2i}-K_{2i}z_{2i}+{M_i}{{\dot{z}}_{\alpha i}} \end{aligned}$$

where $M_{s i}=C_i(\upsilon _i)\upsilon _i+D_i(\upsilon _i)\upsilon _i+g_i$. The term $N_{ci}$ is defined as the following expression

40 $$\begin{aligned} N_{ci}=\Theta _i z_{fi}+\Gamma _{1i} z_{2i}+K_{2i}z_{2i}+(S^{\mathrm {T}}_i)^{+}{\dot{S}}^{\mathrm {T}}_i z_{2i} \end{aligned}$$

Utilizing the mean value theorem, then we obtain

41 $$\begin{aligned} ||N_{ci}||\le {\overline{N}}_{ci}(||z_{si}||)||z_{si}|| \end{aligned}$$

where the bounding function ${\overline{N}}_{ci}(||z_{si}||)$ is a globally positive function. $z_{si}$ is defined as $z_{si}=[z^T_{1i}, z^T_{2i}, S^T_{i}, z^T_{\tau i}, z^T_{fi}]^T$, where $z_{\tau i} \in {{\mathbb {R}}}^{3\times 1}$ denotes

42 $$\begin{aligned} z_{\tau i}=\tau _i(t)-\tau _i(t-t_d)=\int ^t_{t-t_d}{\dot{\tau }}_i(\theta ){\text {d}}\theta \end{aligned}$$

Design the following control law

43 $$\begin{aligned} \tau _{i}(t)=&M_i{\dot{\alpha }}_{ci}+M_{si}+K_{2i}z_{fi}+(S^{\mathrm {T}}_{i})^{+}\left( -{\frac{{{k_{di}}e_{\rho i}^2}}{{k_{ai}^2 - e_{\rho i}^2}} - \frac{{{k_{\psi i}}e_{\psi i}^2}}{{k_{bi}^2 - e_{\psi i}^2}}}+{M_i}{{\dot{z}}_{\alpha i}}+ z_{\alpha i}^{\mathrm {T}}\Theta _{1i}-z_{2 i}^{\mathrm {T}}\Theta _{1i}\right) \end{aligned}$$

### **Theorem 1**

*Consider N+1 USVs with dynamics* (1) *and* (2) *satisfying Assumptions* 1–3, *under the virtual control laws* (27), (28) *and* (29), *the filter* (30) *and control input* (43). *For any* $\Omega _0>0$ *for the initial conditions* $V_{2i}(0)<\Omega _0$, *then the following properties hold*

- *The connectivity and the collision-free are preserved for a pair of leader and follower in the formation design.*
- *The tracking errors converge to neighbour around zero and all signals are uniformly ultimately bounded.*
- *The leader-follower platoon can be achieved in the presence of input time delay.*

### *Proof of Theorem 1*

The quadratic form Lyapunov–Krasovskii candidate is chosen as

44 $$\begin{aligned} V_{2i}=&V_{1i}+\displaystyle \frac{1}{2}z_{2i}^{\mathrm {T}}z_{2i} +\displaystyle \frac{1}{2}S^{\mathrm {T}}_iM_iS_i +\displaystyle \frac{1}{2}z_{fi}^{\mathrm {T}}z_{fi}+\upsilon _i\int ^t_{t-t_d} \left(\int ^t_{w}||\dot{\tau _i}(\theta )||^2{\text {d}}\theta \right) {\text {d}}w+\displaystyle \frac{1}{2}z_{\alpha i}^{\mathrm {T}}z_{\alpha i} \end{aligned}$$

Suppose $\Omega _i$ is the maximum value of ${\dot{\alpha }}_{i}$ and let $B_i=A_i$. Differentiating $V_{2i}$ and according to (35), (36), (38) and (39), we have

45 $$\begin{aligned} {\dot{V}}_{2i}=&{\dot{V}}_{1i}+z_{2i}^{\mathrm {T}}{\dot{z}}_{2i}+S^{\mathrm {T}}_i M_i\dot{S_i}+z_{fi}{\dot{z}}_{fi}+\upsilon _i t_d||\dot{\tau _i}(\theta )||^2-\upsilon _i\int ^t_{t-t_d} ||\dot{\tau _i}(\theta )||^2{\text {d}}\theta +z_{\alpha i}^{\mathrm {T}}{\dot{z}}_{\alpha i}\nonumber \\ =&- {k_{di}}e_{\rho i}^2 - {k_{\psi i}}e_{\psi i}^2 + z_{\alpha i}^{\mathrm {T}}\Theta _{1i}-z_{2 i}^{\mathrm {T}}\Theta _{1i}-A_iz^2_{\alpha i}+|z_{\alpha i}||\Omega _i|+z_{2i}^{\mathrm {T}}(\dot{S_{i}}\nonumber \\&-M_i^{-1}(\tau _i(t-t_d)-\tau _i(t))+K_{2i} S_{i}-\Theta _{i} z_{fi}\nonumber \\&-\Gamma _{1i}z_{2i})+S^{\mathrm {T}}_{i}(M_i{\dot{\alpha }}_{ci}+M_{si} -d_i+N_{ci}-\tau _i(t)-K_{2i}S_{i}-K_{2i}z_{2i}-(S^{\mathrm {T}}_{i})^{+} {\dot{S}}^{\mathrm {T}}_{i}z_{22}+{M_i}{{\dot{z}}_{\alpha i}})\nonumber \\&+z^{\mathrm {T}}_fK_{2i}S_{i}-z^{\mathrm {T}}_{fi} \Theta _{i} z_{fi}-z_{fi}^{\mathrm {T}}\Gamma _{1i}z_{2i}+\upsilon _i t_d||{\dot{\tau }}(\theta )||^2-\upsilon _i\int ^t_{t-t_d}||{\dot{\tau }}_i(\theta )||^2 {\text {d}}\theta \nonumber \\ =&- {k_{di}}e_{\rho i}^2 - {k_{\psi i}}e_{\psi i}^2 + z_{\alpha i}^{\mathrm {T}}\Theta _{1i}-z_{2 i}^{\mathrm {T}}\Theta _{1i}-A_iz^{2}_{\alpha i}+|z_{\alpha i}||\Omega _i|-z_{2i}^{\mathrm {T}}\Gamma _{1i}z_{2i}\nonumber \\&+z_{2i}^{\mathrm {T}}M_i^{-1}z_{\tau i}-z_{2i}^{\mathrm {T}}(\Gamma _{1i}+I)z_{fi}-S^{\mathrm {T}}_{i} K_{2i} S_{i}\nonumber \\&+S^{\mathrm {T}}_{i}\big [M_i{\dot{\alpha }}_{ci}+M_{si}-d_i+N_{ci}-\tau _i(t) +{M_i}{{\dot{z}}_{\alpha i}}\big ]+z_{fi}^{\mathrm {T}}K_{2i}S_{i}-z_{fi}^{\mathrm {T}} \Theta _i z_{fi}+\upsilon _i t_d||{\dot{\tau }}_i(\theta )||^2\nonumber \\&-\upsilon _i\int ^t_{t-t_d}|| {\dot{\tau }}_i(\theta )||^2{\text {d}}\theta \end{aligned}$$

Substituting (43) into (45) and considering Assumption 3, we obtain

46 $$\begin{aligned} {\dot{V}}_{2i}=&-{k_{di}}e_{\rho i}^2 - {k_{\psi i}}e_{\psi i}^2 -{\frac{{{k_{di}}e_{\rho i}^2}}{{k_{ai}^2 - e_{\rho i}^2}} - \frac{{{k_{\psi i}}e_{\psi i}^2}}{{k_{bi}^2 - e_{\psi i}^2}}} -z_{2i}^{\mathrm {T}}\Gamma _{1i}z_{2i}-S^{\mathrm {T}}_{i} K_{2i}S_{i}-z_{fi}^{\mathrm {T}} \Theta _{i} z_{fi}\nonumber \\&+\,z_{2i}^{\mathrm {T}}M_i^{-1}z_{\tau i}-z_{2i}^{\mathrm {T}}(\Gamma _{1i}+I)z_{fi}\nonumber \\&+\,S^{\mathrm {T}}_{i}N_{ci}-S^{\mathrm {T}}_{i}d_i+\upsilon _i t_d||{\dot{\tau }}_i(\theta )||^2 -\upsilon _i\int ^t_{t-t_d}||{\dot{\tau }}_i(\theta )||^2{\text {d}}\theta +\frac{z^2_{\alpha i}}{2}+\frac{\Omega ^2_{ i}}{2}-A_iz^2_{\alpha i}\nonumber \\ \le&-{k_{di}}e_{\rho i}^2 - {k_{\psi i}}e_{\psi i}^2 -{\frac{{{k_{di}}e_{\rho i}^2}}{{k_{ai}^2 - e_{\rho i}^2}} - \frac{{{k_{\psi i}}e_{\psi i}^2}}{{k_{bi}^2 - e_{\psi i}^2}}}-\lambda _{{\text {min}}}(\Gamma _{1i})z^{\mathrm {T}}_{2i}z_{2i} -S^{\mathrm {T}}_{i}K_{2i}S_{i}-\lambda _{{\text {min}}}(\Theta _{i})z_{fi}^{\mathrm {T}}z_{fi}\nonumber \\&+\,\overline{M_i^{-1}}||z_{2i}||||z_{\tau i}||+\overline{(-\Gamma _{1i}-I)}||z_{2i}||||z_{fi}||+{\overline{N}}_{c i}(||z_{si}||)||z_{si}||||S_{i}||+\overline{d_i}||S_{i}||+\upsilon _i t_d||{\dot{\tau }}_i(\theta )||^2\nonumber \\&-\,\upsilon _i\int ^t_{t-t_d}||{\dot{\tau }}_i(\theta )||^2{\text {d}}\theta +\frac{z^2_{\alpha i}}{2}+\frac{\Omega ^2_{ i}}{2}-A_iz^2_{\alpha i} \end{aligned}$$

Based on the Young’s inequality, the term ${\overline{N}}_{ci}(||z_{si}||)||z_{si}||||S_i||$ in (46) yields

47 $$\begin{aligned}&{\overline{N}}_{ci}(||z_{si}||)||z_{si}||||S_i||\le \displaystyle \frac{\sigma _{3i}}{4}{\overline{N}}^2_{ci}(||z_{si}||)||z_{si}||^2 +\displaystyle \frac{1}{\sigma _{3i}}||S_i||^2\nonumber \\&\quad \le \displaystyle \frac{\sigma _{3i}}{4}{\overline{N}}^2_{ci} \big (||z_{si}||)(||z_{1i}||^2+||z_{2i}||^2+||S_i||^2+||z_{\tau i}||^2+||z_{fi}||^2\big )+\displaystyle \frac{1}{\sigma _{3i}}||S_i||^2 \end{aligned}$$

Moreover, for $||z_{1i}||<||N_{bi}||$, the following inequalities holds

48 $$\begin{aligned}&\displaystyle \frac{\sigma _{3i}}{8}\overline{{N}_{ci}}^2(||z_{si}||) z_{1i}^Tz_{1i}-z_{1i}^Tk_{di}z_{1i} -\displaystyle \frac{ z^T_{1i}k_{di} z_{1i}}{N^T_{ai}I_{x}N_{ai} -z_{1i}^TI_{x}z_{1i}}\nonumber \\&\quad \le -\displaystyle \frac{(\lambda _{{\text {min}}}(k_{di}) -\displaystyle \frac{\sigma _{3i}}{8}{\overline{N}}_{ci}^2(||z_{si}||)) z_{1i}^TI_xz_{1i}}{N^T_{ai}I_{x}N_{ai} -z^T_{1i}I_{x}z_{1i}}\nonumber \\&\quad \le -(\lambda _{{\text {min}}}(k_{di}) -\displaystyle \frac{\sigma _{3i}}{8}{\overline{N}}_{ci}^2(||z_{si}||)) {\text {ln}}\displaystyle \frac{N^T_{ai}I_{x}N_{ai}}{N^T_{ai}I_{x} N_{ai}-z^T_{1i}I_{x}z_{1i}} \end{aligned}$$

and

49 $$\begin{aligned}&\displaystyle \frac{\sigma _{3i}}{8}{\overline{N}}_{ci}^2(||z_{si}||) z_{1i}^Tz_{1i}-z_{1i}^Tk_{\psi i}z_{1i} -\displaystyle \frac{ z^T_1 k_{\psi i} z_{1i}}{N^T_{bi}I_{x}N_{bi}-z_{1i}^TI_{y}z_{1i}}\nonumber \\&\quad \le -\displaystyle \frac{(\lambda _{{\text {min}}}(k_{\psi i}) -\displaystyle \frac{\sigma _{3i}}{8}{\overline{N}}_{ci}^2(||z_{si}||)) z_{1i}^TI_yz_{1i}}{N^T_{bi}I_{y}N_{bi} -z^T_1I_{y}z_{1i}}\nonumber \\&\quad \le -(\lambda _{{\text {min}}}(k_{\psi i}) -\displaystyle \frac{\sigma _{3i}}{8}{\overline{N}}_{ci}^2(||z_{si}||)) {\text {ln}}\displaystyle \frac{N^T_{bi}I_{y}N_{bi}}{N^T_{bi}I_{y}N_{bi} -z_{1i}^TI_{y}z_{1i}} \end{aligned}$$

Then the time derivative of ${V}_{2i}$ yields

50 $$\begin{aligned} {\dot{V}}_{2i}\le&-(\lambda _{{\text {min}}}(k_{di}) -\displaystyle \frac{\sigma _{3i}}{8}{\overline{N}}_{ci}^2(||z_{si}||)) {\text {ln}}\displaystyle \frac{N^T_{ai}I_{x}N_{ai}}{N^T_{ai}I_{x}N_{ai} -z_{1i}^TI_{x}z_{1i}}-(\lambda _{{\text {min}}}(k_{\psi i})\nonumber \\&-\displaystyle \frac{\sigma _{3i}}{8}{\overline{N}}_{ci}^2(||z_{si}||)) {\text {ln}}\displaystyle \frac{N^T_{bi}I_{y}N_{bi}}{N^T_{bi}I_{y}N_{bi} -z_{1i}^TI_{y}z_{1i}}\nonumber \\&-\left[ \lambda _{{\text {min}}}(\Gamma _{1i})-\displaystyle \frac{\sigma _{3i}}{4} {\overline{N}}_{ci}^2(||z_{si}||)\right] z^{\mathrm {T}}_{2i}z_{2i}- \left[ \lambda _{{\text {min}}}(K_{2i})-\displaystyle \frac{\sigma _{3i}}{4} {\overline{N}}_{ci}^2(||z_{si}||)\right] S_i^{\mathrm {T}}S_i\nonumber \\&-\left[ \lambda _{{\text {min}}}(\Theta _{i})-\displaystyle \frac{\sigma _{3i}}{4} {\overline{N}}_{ci}^2(||z_{si}||)\right] z_{fi}^{\mathrm {T}}z_{fi} +\displaystyle \frac{\sigma _{1i}\overline{M_i^{-1}}^2}{4}||z_{2i}||^2 +\displaystyle \frac{\sigma _{2i}\overline{(-\Gamma _{1i}-I)}^2}{4}||z_{2i}||^2\nonumber \\&+\left[ \displaystyle \frac{1}{\sigma _{1i}}+\displaystyle \frac{\sigma _{3i}}{4} \overline{N_{ci}}^2(||z_{si}||)\right] ||z_{\tau i}||^2+\displaystyle \frac{1}{\sigma _{2i}}||z_{fi}||^2 +\displaystyle \frac{1}{\sigma _{3i}}||S_i||^2+\displaystyle \frac{\sigma _{4i}}{4}\overline{d_i}^2\nonumber \\&+\displaystyle \frac{1}{\sigma _{4i}}||S_i||^2+\upsilon _i t_d||{\dot{\tau }}_i(\theta )||^2-\upsilon _i\int ^t_{t-t_d}||{\dot{\tau }}_i (\theta )||^2{\text {d}}\theta +\frac{\Omega ^2_{ i}}{2}-(A_i-\frac{1}{2}I)z^2_{\alpha i} \end{aligned}$$

Cauchy–Schwarz inequality gives the upper bound of $||z_{\tau i}||$ as

51 $$\begin{aligned} ||z_{\tau i}||^2 \le t_d \int ^t_{t-t_d}||{\dot{\tau }}_i(\theta )||^2{\text {d}}\theta \end{aligned}$$

Moreover, it can be proven that

52 $$\begin{aligned} \int ^t_{t-t_d}\bigg [\int ^t_w||{\dot{\tau }}_i(\theta )||^2{\text {d}}\theta \bigg ] {\text {d}}w \le t_d\int ^t_{t-t_d}||{\dot{\tau }}_i(\theta )||^2{\text {d}}\theta \end{aligned}$$

According to (51) and (52), the inequality (50) becomes

53 $$\begin{aligned} {\dot{V}}_{2i}\le&- (\lambda _{{\text {min}}}(k_{di}) -\displaystyle \frac{\sigma _{3i}}{8}{\overline{N}}_{ci}^2(||z_{si}||)) \ln \frac{{k_{ai}^2}}{{k_{ai}^2 - e_{\rho i}^2}} - (\lambda _{{\text {min}}}(k_{\psi i}) -\displaystyle \frac{\sigma _{3i}}{8}{\overline{N}}_{ci}^2(||z_{si}||)) \ln \frac{{k_{bi}^2}}{{k_{bi}^2 - e_{\psi i}^2}}\nonumber \\&-\bigg [\lambda _{{\text {min}}}(\Gamma _{1i})-\displaystyle \frac{\sigma _{1i} \overline{M_i^{-1}}^2}{4} -\displaystyle \frac{\sigma _{3i}}{4}{\overline{N}}_{ci}^2(||z_{si}||) -\displaystyle \frac{\sigma _{2i}\overline{(-\Gamma _{1i}-I)}^2}{4}\bigg ]z^ {\mathrm {T}}_{2i}z_{2i} +\displaystyle \frac{\sigma _{4i}}{4}\overline{d_i}^2+\upsilon _i t_d ||{\dot{\tau }}(\theta )||^2\nonumber \\&-\bigg [\lambda _{{\text {min}}}(K_{2i})-\displaystyle \frac{1}{\sigma _{3i}} -\displaystyle \frac{1}{\sigma _{4i}} -\displaystyle \frac{\sigma _{3i}}{4}{\overline{N}}_{ci}^2(||z_{si}||) \bigg ]S^{\mathrm {T}}_{i}S_{i}-\bigg [\lambda _{{\text {min}}}(\Theta _{i}) -\displaystyle \frac{1}{\sigma _{2i}} -\displaystyle \frac{\sigma _{3i}}{4}{\overline{N}}_{ci}^2(||z_{si}||) \bigg ]z_{fi}^{\mathrm {T}}z_{fi}\nonumber \\&-\bigg [\upsilon _i-\displaystyle \frac{t_d}{\sigma _{1i}}-\displaystyle \frac{t_d\sigma _{3i}}{4}{\overline{N}}_{ci}^2(||z_{si}||)\bigg ] \int ^t_{t-t_d}||{\dot{\tau }}_i(\theta )||^2{\text {d}}\theta +\frac{\Omega ^2_{ i}}{2}-(A_i-\frac{1}{2}I)z^2_{\alpha i}\nonumber \\ \le&- (\lambda _{{\text {min}}}(k_{di}) -\displaystyle \frac{\sigma _{3i}}{8}{\overline{N}}_{ci}^2(||z_{si}||)) \ln \frac{{k_{ai}^2}}{{k_{ai}^2 - e_{\rho i}^2}} - (\lambda _{{\text {min}}}(k_{\psi i}) -\displaystyle \frac{\sigma _{3i}}{8}{\overline{N}}_{ci}^2(||z_{si}||)) \ln \frac{{k_{bi}^2}}{{k_{bi}^2 - e_{\psi i}^2}}\nonumber \\&-\bigg [\lambda _{{\text {min}}}(\Gamma _{1i})-\displaystyle \frac{\sigma _{1i} \overline{M_i^{-1}}^2}{4} -\displaystyle \frac{\sigma _{3i}}{4}{\overline{N}}_{ci}^2(||z_{si}||) -\displaystyle \frac{\sigma _{2i}\overline{(-\Gamma _{1i}-I)}^2}{4}\bigg ]z^{\mathrm {T}}_{2i}z_{2i} +\displaystyle \frac{\sigma _{4i}}{4}\overline{d_i}^2+\upsilon _i t_d|| {\dot{\tau }}_i(\theta )||^2\nonumber \\&-\bigg [\lambda _{{\text {min}}}(K_{2i})-\displaystyle \frac{1}{\sigma _{3i}}- \displaystyle \frac{1}{\sigma _{4i}} -\displaystyle \frac{\sigma _{3i}}{4}\overline{N_{ci}}^2(||z_{s
i}||) \bigg ]S^{\mathrm {T}}_{i}S_{i} -\bigg [\lambda _{{\text {min}}}(\Theta _{i})-\displaystyle \frac{1}{\sigma _{2i}} -\displaystyle \frac{\sigma _{3i}}{4}\overline{N_{ci}}^2(||z_{si}||) \bigg ]z_{fi}^{\mathrm {T}}z_{fi}\nonumber \\&-\bigg [\displaystyle \frac{\upsilon _i}{t_d}-\displaystyle \frac{1}{\sigma _{1i}} -\displaystyle \frac{\sigma _{3i}}{4}\overline{N_{ci}}^2(||z_{si}||)\bigg ] \int ^t_{t-t_d}\bigg [\int ^t_w||{\dot{\tau }}_i(\theta )||^2{\text {d}}\theta \bigg ] {\text {d}}w+\frac{\Omega ^2_{ i}}{2}-(A_i-\frac{1}{2}I)z^2_{\alpha i}\nonumber \\ \le&-\rho _{ci}V_{2i}+\beta _{ci} \end{aligned}$$

where $$\rho _{ci}={\text {min}}\bigg [2(\lambda _{{\text {min}}}(k_{di}) -\displaystyle \frac{\sigma _{3i}}{4}{\overline{N}}_{ci}^2(||z_{si}||)), 2(\lambda _{{\text {min}}}(k_{\psi i})-\displaystyle \frac{\sigma _{3i}}{4}{\overline{N}}_{ci}^2(||z_{si}||)), 2(\lambda _{{\text {min}}}(\Gamma _{1i})-\displaystyle \frac{\sigma _{1i} \overline{M_i^{-1}}^2}{4}-\displaystyle \frac{\sigma _{3i}}{4}{\overline{N}}_{ci}^2 (||z_{si}||) -\displaystyle \frac{\sigma _{2i}\overline{(-\Gamma _{1i}-I)}^2}{4}), 2(\lambda _{{\text {min}}}(K_{2i})-\displaystyle \frac{1}{\sigma _{3i}}- \displaystyle \frac{1}{\sigma _{4i}}-\displaystyle \frac{\sigma _{3i}}{4} {\overline{N}}_{ci}^2(||z_{si}||))/\lambda _{{\text {max}}}(M_i), 2(\lambda _{\text {min}}(\Theta _{i})-\displaystyle \frac{1}{\sigma _{2i}} -\displaystyle \frac{\sigma _{3i}}{4}\overline{N_{ci}}^2(||z_{si}||)), (\displaystyle \frac{1}{t_d}-\displaystyle \frac{1}{\sigma _{1i}\upsilon _i} -\displaystyle \frac{\sigma _{3i}}{4\upsilon _i}\overline{N_{ci}}^2(||z_{si}||)), A_i-\frac{1}{2}I \bigg ]$$, $$\beta _{ci}=\displaystyle \frac{\sigma _{4i}}{4}\overline{d_i}^2+\upsilon _i t_d||{\dot{\tau }}_i(\theta )||^2+\frac{\Omega ^2_{ i}}{2}>0$$.

If the tuning parameters are selected as

54 $$\begin{aligned}&\lambda _{\text {min}}(k_{di})>\displaystyle \frac{\sigma _{3i}}{4}{\overline{N}}_{ci}^2 (||z_{si}||) \end{aligned}$$

55 $$\begin{aligned}&\lambda _{\text {min}}(k_{\psi i})>\displaystyle \frac{\sigma _{3i}}{4}{\overline{N}}_{ci}^2(||z_{si}||) \end{aligned}$$

56 $$\begin{aligned}&\lambda _{\text {min}}(\Gamma _{1i})+\displaystyle \frac{\sigma _{2i} \overline{(-\Gamma _{1i}-I)}^2}{4}>\displaystyle \frac{\sigma _{1i} \overline{M_i^{-1}}^2}{4}+\displaystyle \frac{\sigma _{3i}}{4}{\overline{N}}_{ci}^2 (||z_{si}||) \end{aligned}$$

57 $$\begin{aligned}&\lambda _{\text {min}}(K_{2i})>\displaystyle \frac{1}{\sigma _{3i}} +\displaystyle \frac{1}{\sigma _{4i}}+\displaystyle \frac{\sigma _{3i}}{4} {\overline{N}}_{ci}^2(||z_{si}||) \end{aligned}$$

58 $$\begin{aligned}&\lambda _{\text {min}}(\Theta _{i})>\displaystyle \frac{1}{\sigma _{2i}} +\displaystyle \frac{\sigma _{3i}}{4}{\overline{N}}_{ci}^2(||z_{si}||) \end{aligned}$$

59 $$\begin{aligned}&\displaystyle \frac{\upsilon _i}{t_d}>\displaystyle \frac{1}{\sigma _{1i}} +\displaystyle \frac{\sigma _{3i}}{4}{\overline{N}}_{ci}^2(||z_{si}||) \end{aligned}$$

60 $$\begin{aligned}&A_i>\frac{1}{2}I \end{aligned}$$

then $\rho _{ci}>0$. It is obvious that $V_{i}(t)$ is semi-global uniformly ultimate boundedness for $V_i(0)\le B_{0i}$, where $B_{0i}=V_i(\epsilon _{1},z_{2i}, S_{i} ,z_{\tau },z_{fi})$ is a positive constant. $\square$

## Simulation results

To demonstrate the performance of the proposed formation control method, a platoon consisting of one leader and three followers is designed. The marine vehicle model parameters are taken from Cybership-II^31^. It is a 1:70 scale replica of a supply vessel from the marine control laboratory in Norwegian University of Science and Technology. The corresponding parameters are listed in Table 1.

**Table 1:** Parameters of the model vessel.

$$m_i$$	23.800	$$Y_{v_i}$$	$$-$$ 0.8897	$$N_{v_i} $$	0.0313	$$X_{{\dot{u}}_i}$$	$$-$$ 2.000
$$I_{z_i} $$	1.7600	$$Y_{|v_i|v_i}$$	$$-$$ 36.4729	$$N_{|v_i|v_i}$$	3.9565	$$Y_{{\dot{v}}_i}$$	$$-$$ 10.0000
$x_{gi}$	0.0460	$Y_{|r_i|v_i}$	$-$ 0.8050	$N_{|r_i|v_i}$	0.1300	$Y_{{\dot{r}}_i}$	0
$$X_{u_i} $$	$$-$$ 0.7225	$$Y_{r_i}$$	$$-$$ 7.2500	$$N_{ri}$$	$$-$$ 1.900	$$N_{{\dot{v}}i}$$	0
$$X_{|u_i|u_i} $$	$$-$$ 1.3274	$$Y_{|v_i|r_i}$$	$$-$$ 0.8450	$$N_{|v_i|r_i}$$	0.0800	$$ N_{{\dot{r}}_i}$$	$$-$$ 1.0000
$X_{u_iu_iu_i}$	$-$ 5.8664	$Y_{|r_i|r_i}$	$-$ 0.4500	$N_{|r_i|r_i}$	$-$ 0.7500

**Figure 2:**
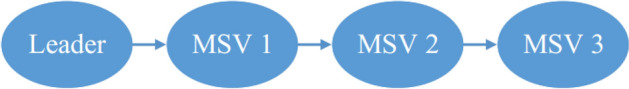
Communication graph among the 4 MSVs.

The communication relationship of the 4 MSVs is shown in Fig. 2. The desired distance between each two marine vessels is considered as 5 m. The initial positions of each MSV are ${\eta _0} = {\left[ {0,0,0} \right] ^{\mathrm {T}}}$, ${\eta _1} = {\left[ {0,5,0} \right] ^{\mathrm {T}}}, {\eta _2} = {\left[ {0,10,0} \right] ^{\mathrm {T}}}, {\eta _3} = {\left[ {0,15,0} \right] ^{\mathrm {T}}}$ and their initial velocities are ${\upsilon _i} = {\left[ {0,0,0} \right] ^{\mathrm {T}}}$. The input delay time is 2s. The control parameters are chosen as $k_{d1}=12, k_{d2}=k_{d3}=1, k_{\psi 1}=1, k_{\psi 2}=k_{\psi 3}=0.5, k_{a1}=k_{a2}=k_{a3}=1, k_{b1}=k_{b1}=k_{b1}=0.5, K_{2i}={\mathrm {diag}}[6,6,4], \Gamma _{1i}={\mathrm {diag}}[0.001,0.001,0.001], \Theta _i={\mathrm {diag}}[0.1,0.1,0.1], A_{i}=B_{i}=U_{i}={\mathrm {diag}}[8,8,6]$.

**Figure 3:**
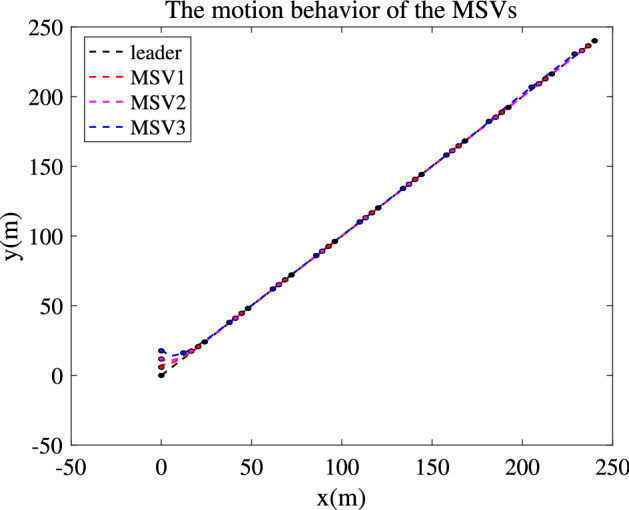
Platoon formation process of the 4 MSVs.

**Figure 4:**
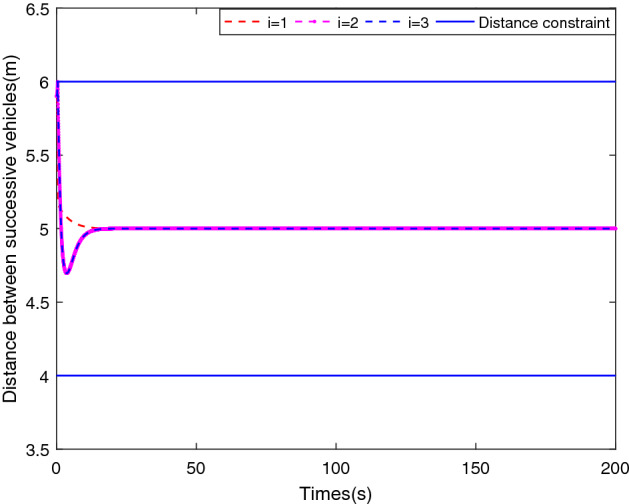
Evolution of LOS ranges along with the predefined boundaries.

**Figure 5:**
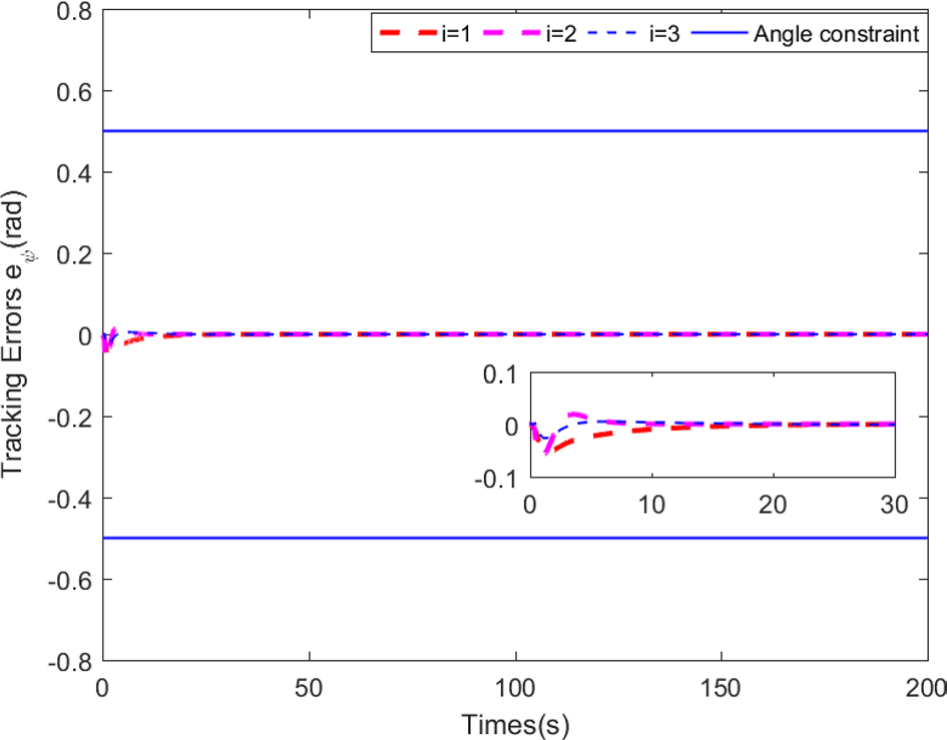
Bearing angle tracking errors along with predefined boundaries.

**Figure 6:**
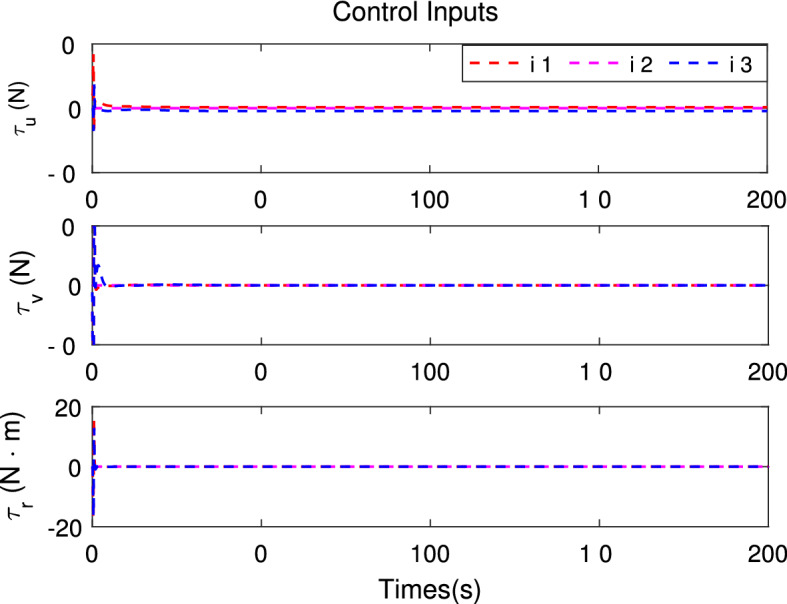
Time response of the control inputs.

**Figure 7:**
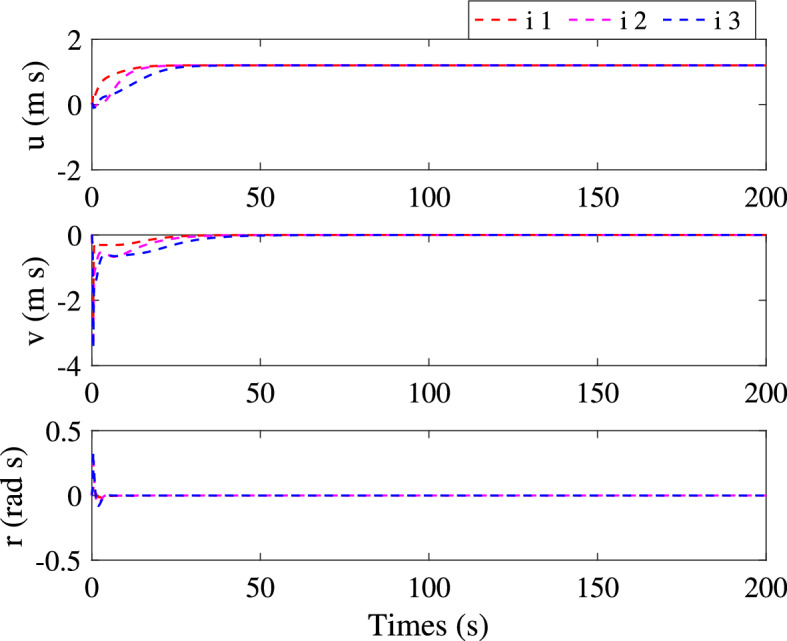
Velocities of three followers.

Simulation results are shown in Figs. 3, 4, 5, 6 and 7. The response curves of MSV1, MSV2 and MSV3 are plotted in red dash lines, purple dash lines, and blue dash lines, respectively. Figure 3 depicts platoon formation process of the 4 MSVs. Each marine vessel follows its leader with a satisfactory tracking performance during the entire process of moving. The distances between successive vehicles shown in Fig. 4 stay within the maximum connectivity distance 6 m and the minimum collision distance 4 m. It indicates that the LOS range tracking errors are within the predefined region bound. The desired distances between each leader and follower satisfy the inequality constraints $0<\ {\rho _{i,\min col}} < {\rho _i} \le {\rho _{i,\max com}}, i=1,2,3$. The connectivity and collision prevention among the 4 MSVs are guaranteed during the formation achievement. Figure 5 represents the bearing angle tracking errors $e_\psi$, which does not violate the constraints $[-0.5{\mathrm {rad}}, 0.5{\mathrm {rad}}]$. It demonstrates that bearing angles are constrained effectively. Figure 6 displays the control inputs of the following MSVs. The velocities of three followers are shown in Fig. 7. The simulation results demonstrate the connectivity preservation and collision prevention are satisfied, and the string of the MSVs can achieve the formation tracking in the presence of input delay.

## Conclusions

Platoon formation control for a string MSVs has been developed in the presence of input delay and output constraints in this paper. BLF has been proposed to constraint LOS range and bearing angle tracking errors to satisfy the collision avoidance and connectivity maintenance. Next, bioinspired neurodynamics has been incorporated into the kinematic design to avoid the complicated computation of the leader vessel’s acceleration. Furthermore, input delay system has been converted into a delay-free system and the stability has been proven by Lyapunov–Krasovskii analysis. The signals of the closed-loop platoon system were uniformly ultimate boundedness by regulating the appropriate parameters. Finally, the simulation results have demonstrated the effectiveness of the proposed control method.
